# Physiological evidence of sensory integration in the electrosensory lateral line lobe of *Gnathonemus petersii*

**DOI:** 10.1371/journal.pone.0194347

**Published:** 2018-04-11

**Authors:** Sylvia Fechner, Kirsty Grant, Gerhard von der Emde, Jacob Engelmann

**Affiliations:** 1 University of Bonn, Institute for Zoology, Bonn, Germany; 2 UNIC, CNRS, 1 Avenue de la Terrasse, Gif-sur Yvette, France; 3 University of Bielefeld, Biology – AG Active Sensing, Bielefeld, Germany; McGill University Department of Physiology, CANADA

## Abstract

Mormyrid fish rely on reafferent input for active electrolocation. Their electrosensory input consists of phase and amplitude information. These are encoded by differently tuned receptor cells within the Mormyromasts, A- and B-cells, respectively, which are distributed over the animal’s body. These convey their information to two topographically ordered medullary zones in the electrosensory lateral line lobe (ELL). The so-called medial zone receives only amplitude information, while the dorsolateral zone receives amplitude and phase information. Using both sources of information, Mormyrid fish can disambiguate electrical impedances. Where and how this disambiguation takes place is presently unclear. We here investigate phase-sensitivity downstream from the electroreceptors. We provide first evidence of phase-sensitivity in the medial zone of ELL. In this zone I-cells consistently decreased their rate to positive phase-shifts (6 of 20 cells) and increased their rate to negative shifts (11/20), while E-cells of the medial zone (3/9) responded oppositely to I-cells. In the dorsolateral zone the responses of E- and I-cells were opposite to those found in the medial zone. Tracer injections revealed interzonal projections that interconnect the dorsolateral and medial zones in a somatotopic manner. In summary, we show that phase information is processed differently in the dorsolateral and the medial zones. This is the first evidence for a mechanism that enhances the contrast between two parallel sensory channels in Mormyrid fish. This could be beneficial for impedance discrimination that ultimately must rely on a subtractive merging of these two sensory streams.

## Introduction

Neuronal maps in sensory physiology have been studied from at least two perspectives, defining a map as a neuronal representation that is based on the topography of the receptor array and/or as a topographic neuronal representation of features that are computed independently from the topography of the receptor array. The latter are considered as evidence that neuronal maps can be beneficial beyond the idea of optimal wiring or developmental constraints. To which degree neuronal maps are of functional relevance is still unanswered [1–3]. It is commonly accepted that topographic representations facilitate localisation of spatially sparse inputs [4], but no optimal representation of multidimensional inputs in the low-dimensional space of neurons has been defined yet [5–8].

Parallel (mapped) processing of sensory features is common at early processing stages, particularly at the level of primary sensory input. This has been studied extensively in the electrosensory lateral line lobe (ELL) of weakly-electric fishes. These fish can actively generate an electric field through the discharge of their electric organ (EOD). The environmental modulations of this field are encoded through an array of cutaneous electroreceptors. This input is used for active electrolocation [9]. Research on the ELL of Gymnotiform fish has advanced our understanding of the cellular mechanisms that aid in extracting different features from a single sensory stream through parallel processing [10]. Here, three parallel topographic maps exist in which subpopulations of neurons with differing spatiotemporal tuning properties process the information of the sensory input from a single class of electroreceptors in parallel. The three ELL maps likely evolved through duplication of a plesiomorphic ampullary or mechanosensory lateral line map [11] and have been interpreted as adaptations to the increased behavioural repertoire that electroreception offered these fishes [12]. The alternative option to add the new computational loads to the existing neuronal architecture apparently led to significant constraints, thus favouring a duplication of maps [13–16]. Interestingly, the three maps in the Gymnotiform ELL lack interconnections [13]. At the midbrain their input converges on multiple ill-defined maps [10] but it is unclear if the input of the maps converges on the single cell level [13]. Recently it was shown that neurones in the midbrain can extract specific features of the sensory input that are not being responded to at the earlier levels of the sensory pathway. This gives support to the notion that convergence of parallel sensory streams can enable the extraction of specific sensory cues [17–19].

The second family of weakly electric fish, the Mormyridae, allow the investigation of parallel processing of features that are already separated at the receptor level. This offers the potential to unravel how merging of parallel sensory streams can aid in the extraction of behaviourally relevant computed sensory features. Mormyrid electroreceptors (Mormyromasts) are sensitive to amplitude and waveform modulations of the electric field [20]. Contrary to Gymnotiformes, two differently tuned sensory cells in each mormyromast, A- and B-cells, respectively, are responsible for encoding these features [21]. Afferents of A- and B-cells respond to an increase in the amplitude of the EOD with a decrease of their first-spike latency and an increase in spike number [22,23]. B-cell afferents in addition are responsive to the waveform distortions caused by capacitive objects [24,25]. Amplitude and waveform modulations (phase) thus can be considered as two parallel streams of sensory information. As capacitive and resistive properties of an object can modify the responses in the B-cells, whereas the A-cells are tuned to the resistive properties only, a direct separation of resistive and capacitive properties is impossible. This requires a central (subtractive) comparison of both sensory streams [24]. A series of behavioural studies showed that *Gnathonemus* can indeed discriminate between resistive and capacitive properties unequivocally [26,27] and this has further strengthened the hypothesis that the parallel sensory streams of A- and B-cell input need to be merged centrally [24,28].

A- and B-cell information is processed in the somatotopically organized medial zone (MZ) and the dorsolateral zone (DLZ) [29] of the ELL. Interzonal connections connect the two zones homotopically, preserving the topography between zones [30]. Such connections could aid in the disambiguation of electric phase and amplitude, rendering neurones of the DLZ sensitive to phase only. However, experiments directly addressing this hypothesis found no evidence that neurones in the DLZ are sensitive to phase only, nor support for an acquired waveform-sensitivity in the MZ [31]. Stirred by a recent study that showed that B-cell information is overrepresented in the dorsolateral map of the ELL for the head and chin appendix regions [32], we decided to re-investigate this. Given that the initial study by von der Emde and Bell (1994) considered neurones receiving input from the trunk region only, we speculated that a separation of phase and amplitude might be restricted to the head and chin appendix region. Hence extracellular single-cell recordings and tracer injections were carried out in the rostral parts of the DLZ and MZ, while stimulating neurones with artificially modified EODs that only differed in either waveform or amplitude. We found that phase shifted EODs influenced the neuronal responses in both zones, strongly suggesting that phase information must be conveyed to the medial zone. We further show that responses of phase-sensitive neurones in the DLZ and in the MZ differed in a consistent and zone-specific manner. This differential responsiveness, most likely mediated by the interzonal connections, results in contrast enhancement that would be beneficial for the proposed subtractive mechanism required to discriminate amplitude and phase information.

## Material and methods

### General

A total of 17 individuals of the species *G*. *petersii* were used in the experiments (8.0–12.5 cm in standard length). The fish were acquired from a local supplier (Aquarium Glaser, Frankfurt/Main, Germany) and were kept in groups in 250 l aquaria at 25–27 °C on daily 12/12 h light/dark cycle (water conductivity 90–120 μS cm^-1^).

### Surgery

Surgery was conducted as previously described [33,34]. Briefly, fish were anaesthetised with 0.1 g l^-1^ tricaine (MS-222, Acros organics) followed by an intramuscular injection of 0.3–0.5 μl g^-1^ body weight pancuronium bromide (Roth). The fish was placed on a Styrofoam platform and respirated artificially (MS-222: 0.03 g l^-1^). Before removing dorsal parts of the skin and skull to expose the valvula cerebelli, the skin was locally anaesthetised (Xylocaine gel 2%, AstraZeneca, Wedel, Germany). After surgery, the respiration was switched to fresh aerated water and the Styrofoam platform was removed so that the fish was only held by a plastic rod attached to the skull.

### Ethical statement

The study was carried out in accordance with the recommendations in the Guide for the Care and Use of Laboratory Animals of the National Institutes of Health, with the council of Europe Treaty ETS 123 as well as with the current local laws of Germany where the experiments were performed. The protocol was approved by the Landesamt für Natur, Umwelt und Verbraucherschutz Nordrhein-Westfalen (LANUV, permit 50.203.2-BN7/107). All surgery was performed under sodium bicarbonate buffered tricaine methanesulfonate anaesthesia; in addition local analgesia was administered at the wound margins (lidocaine), and all efforts were made to minimize suffering. Monitoring of animal welfare during the experiments was conducted (see [35]). When fish ceased to produce EODs for 5 minutes or when their EOD rate became highly irregular (mean EOD rate / standard deviation < 2, values obtained for recent 10 minutes) these were taken as indicators for humane endpoint criteria. These criteria were not fulfilled for any animal in our study.

### Stimulus generation

In non-curarized fish, the EOD is driven by a descending spino-motor volley known as the EOD motor-command signal (EODC). This descending command continues spontaneously in curarized fish without, however, initiating an EOD. The EODC was recorded with a silver wire bent around the fish’s tail. The EODC then was used to trigger an artificial EOD stimulus (Wavetek, Model 395) delivered at a delay after the first negative peak of the EODC (defined as “time zero”; t_0_). This delay was adjusted to match the fish’s natural EODC/EOD interval, which was recorded before curarisation (between 2.5 and 4.9 ms).

**Fig 1:**
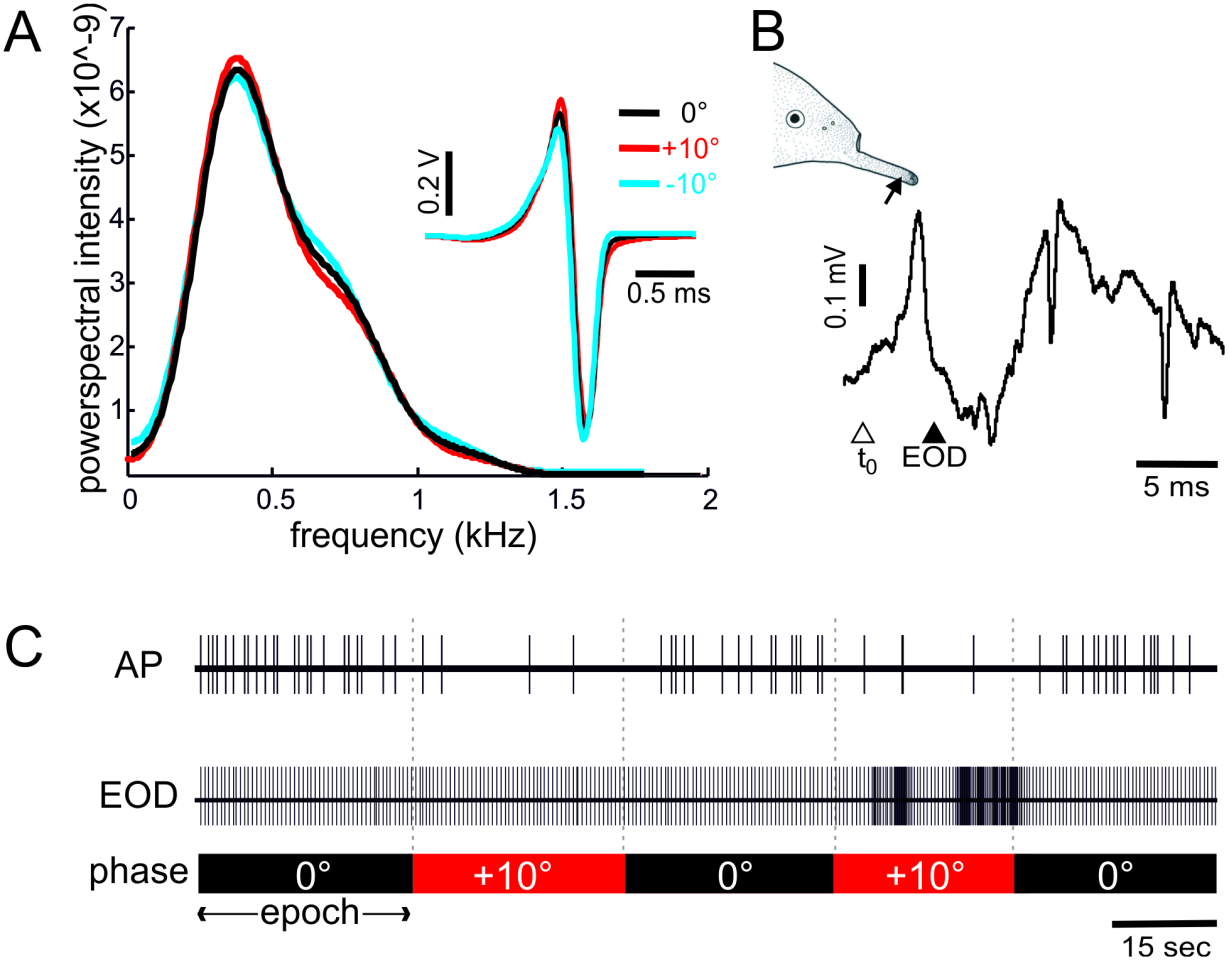
Confirmation of stimuli and schematic stimulus protocol. **A-C: A**. Power-spectra of the EODs used as stimuli. For this analysis the EODs were measured next to the tip of the chin appendix of an animal. Single EODs for the three conditions (0, + and—10°) are shown to the right. Note the differences in the positive-to-negative peak ratios and the similarity of the power spectra. **B**. Exemplary local field potential recorded in the plexiform layer of the medial zone of the ELL in the absence of sensory stimulation. The open triangle below the field potential recording indicates the t_0_ reference, while the filled triangle indicates the time when the EOD would have occurred under natural conditions, i.e. the time at which the artificial stimulus was presented. The schematic indicates the receptive field centre of the encountered cell. **C**. Example of the stimulus protocol. From top to bottom: spikes (AP), EODs and phase of the stimulus. For positive and negative phase shifts two consecutive phase shifts were presented. Each epoch lasted for 30 seconds.

Phase-shifted (+10° or -10°) and unaltered (0°) artificial EODs were used as stimuli. Phase-shifted EODs were constructed by retarding the phase angle of all positive frequencies of the FFT phase spectrum of a pre-recorded EOD by a constant angle while the negative frequencies were advanced by the same angle [36,37]. The time domain of these phase shifted EODs was obtained through an inverse FFT. This ensured that all signals were similar in peak-to-peak amplitude and power spectra. This is in contrast to natural capacitive objects, which induce phase shifts in the range of 0° to -25° and additionally result in changes of the power spectrum and the peak-to-peak amplitude. Thus, only B-cells of the mormyromasts should give differential responses to the phase shifted signals used here [24]. Stimuli were delivered through an isolated symmetry amplifier (Elektronikwerkstatt, Bonn) to a pair of silver electrodes (exposed diameter 2 mm, 1cm apart). This enabled us to stimulate small areas of the fish’s skin. The stimulation electrode was movable and positioned perpendicular to the skin. Measuring the delivered EODs directly at the skin of the animal [38] showed that EODs remained similar in their power spectra and peak-to-peak amplitudes, differing only in their peak ratios (Fig 1).

### Data recording

The motor-command signal was pre-amplified and passed through an amplifier/filter unit (amplification x100, 10 Hz high-pass, 10 kHz low-pass, 50 Hz notch-filter, Elektronikwerkstatt, Uni Bonn). Single cells in different layers of the ELL were recorded extracellularly using glass microelectrodes (resistance 0.8–3.1 MΩ) filled with 3 M NaCl. Field potentials and single cell recordings were amplified (DAM 80; WPI), band pass filtered (1Hz– 1kHz for field potentials; 300Hz-3kHz for single cell recordings) and notch filtered (HumBug; Quest Scientific). All signals were displayed on an oscilloscope (DL 1540 CL; Yokogawa), digitised (10kHz; Power 1401; CED) and stored on a computer. The stereotyped electric-organ corollary discharge (EOCD) evoked field potentials were used as landmarks for determining from which layer and zone of the ELL we recorded [33].

### Stimulus protocol

After establishing a cell’s receptive field, the stimulus electrode was positioned in the centre of the receptive field at a lateral distance of 2 mm from the electroreceptor. To classify the cells, we recorded their response to the EOCD alone as well as to a local stimulus that was time-locked to the natural EOD timing (Fig 1C). This local stimulus was presented using at least five different stimulus amplitudes. For subsequent tests the stimulus amplitude was set slightly above a given cell’s threshold. We distinguish between E- and I-cells: I-cells give a burst of spikes in the absence of sensory stimuli and the number of spikes in the burst decreases with increasing stimulus intensity, while the spike latency increased. E-cells give a burst of spikes to a local sensory stimulus in the centre of their receptive field, but are mostly inactive in the absence of sensory stimuli. An increase of stimulus amplitude causes an increase of spike rate and a decrease in spike latency.

The effect of phase-shifted EODs was tested by stimulating with a basal EOD of zero phase shift and EODs that were shifted by either +10° or -10° (referred to as Φ in the following). Within a trial we switched between the basal and phase-shifted condition twice, starting and ending with the basal condition. Each trial thus consisted of 5 epochs, each lasting 30 seconds, with 2 phase-shifted epochs of either -10° or +10° (see Fig 1C). Negative phase shifts were tested prior to positive phase shift. If recordings were stable, this protocol was repeated with different EOD amplitudes.

### Data analysis

To compare if responses within a trial differed between conditions, i.e. between basal and phase-shifted epochs, we compared the spikes per EOD between epochs (Kruskal-Wallis test followed by Dunn’s post-hoc test, Matlab 11b). A nonparametric test was used as the number of spikes per EOCD-cycle deviated from the normality assumption in some epochs (Kolmogorov Smirnov test, SPSS 14). When a phase shifted epoch significantly differed from at least two of the three zero-phase epochs within a trial we classified this as a reproducible phase-sensitivity. In the Results section we report the number of all cells tested in a given condition followed by the number of cells that were classified as reproducible (N all / N reproducible).

To further analyse the effect of phase shifts per trial we obtained the mean number of spikes per EOD for the un-shifted epochs (*AP*_*basal*_) and the mean number of spikes per EOD for the phase-shifted epochs (*AP*_*shifted*_). From these we calculated the mean number of spikes per EOD and stimulus condition (${\bar{AP}}_{\Phi }=1/n\sum_{1}^{n}AP_{shifted}$, ${\bar{AP}}_{0{}^{\circ}}=1/n\sum_{1}^{n}AP_{basal}$, with Φ being either + or -10°). To quantify how responses differed between basal and phase-shifted conditions within a trial, we calculated the mean rate-difference between the shifted and basal condition $\left(\Delta_{\Phi }={\bar{AP}}_{\Phi }-{\bar{AP}}_{0{}^{\circ}}\right)$. Mean rate-differences for positive shifts will be presented as Δ_+10_ and as Δ_−10_ for negative phase-shifts. First spike latency was analysed in a similar manner and was expressed relative to time zero (t_0_) of the EODC. To compare the effect of phase shifts between I- and E-cells as well as between the DLZ and MZ zone, we used the difference of the mean spike rates per group (one-way ANOVA followed by a Tukey’s test, SPSS 14).

### Anatomy

Different tracers were injected iontophoretically (4–5 μA DC current, 30 minutes, changing of polarity every 5 minutes) at identified layers (ganglionic, plexiform or granular layer) of the MZ or the DLZ during the electrophysiological experiments. Electrodes (resistance < 0.6 MΩ) were filled with biocytin (4% in 3M NaCl), neurobiotin (4% in 3 M NaCl), or a fluorescent dye (Fluoro-Ruby D-1817, 10 kDa, Invitrogen). After a survival time of 10–28 hours, the animals were deeply anaesthetised (MS-222) and perfused with 2% paraformaldehyde and 2% glutaraldehyde in phosphate buffer (0.1 M, pH 7.4).

Serial sections were cut on a vibratome (Vibratome^®^ 1500, TSE systems; Leica 2000, Leica, 80 μm). Injections of biocytin were developed with ABC-complex (Vectastain^®^, ABC Kit, PK-4000; Vector Laboratories) and DAB (3,3’-Diaminobenzine) to reveal labelling. The sections were mounted on glass slides (Thermo scientific Superfrost Plus^™^, Fischer Scientific, Illkirch, France) and counterstained with neutral red. In some sections an additional fluorescent Nissl-stain was applied (NeuroTrace 530, Invitrogen). All fluorescent slices were mounted with Vectashield^®^ (Vector Laboratories, Inc., H-1500).

## Results

As detailed in the introduction phase is represented in an ambiguous manner in the afferent stream, yet Mormyrids can evaluate phase and amplitude independently [26]. However, no evidence for convergence between both sensory streams has been found at the level of the ELL in previous works [20,31]. As the medial and the dorsolateral maps of the ELL are interconnected [39] and phase information is overrepresented in the part of the DLZ map that receives input from the *foveal* chin appendix [32], this lack of interzonal processing in the ELL is surprising. We here thus specifically investigated if interzonal processing of phase information occurs in those regions of the ELL that receive input from the foveal areas. We report data from 41 cells (medial zone = 29 cells, dorsolateral zone = 12 cells, see Table 1) and demonstrate that cells in the medial map respond to phase-shifted stimuli.

**Table 1:** Summary of effects of phase-shifted stimuli on E- and I-cells of DLZ & MZ.

***Dorsolateral zone***				
	**E-cells**		**I-cells**	
	mean change in rate ± std	cell count	mean change in rate ± std	cell count
**Δ**_**+10** all_	-0.76 ± 0.63	9	0.96 ± 1.57	5
**Δ**_**+10** reproducible_	-1.17 ± 0.66	6	1.71 ± 2.06	3
**Δ**_**-10** all_	0.85 ± 0.67	9	-0.49 ± 0.34	5
**Δ**_**-10** reproducible_	1.47 ± 0.74	7	-0.74 ± 0.04	3
***Medial zone***				
	**E-cells**		**I-cells**	
	mean change in rate ± std	cell count	mean change in rate ± std	cell count
**Δ**_**+10** all_	0.30 +/- 0.51	9	-0.40 ± 0.39	20
**Δ**_**+10** reproducible_	0.91 ± 0.47	3	-0.65 ± 0.46	11
**Δ**_**-10** all_	-0.24 +/- 0.24	9	0.22 ± 0.28	20
**Δ**_**-10** reproducible_	-0.52 ± 0.20	3	0.63 ± 0.30	6

Mean change in firing rate with respect to zero phase shifted firing rate for the DLZ and MZ maps separated between E- and I-cells and positive and negative phase shifts. Data is presented for all cells followed by cells with significant and reproducible effects.

### Responses to phase-shifts in the DLZ

**Fig 2:**
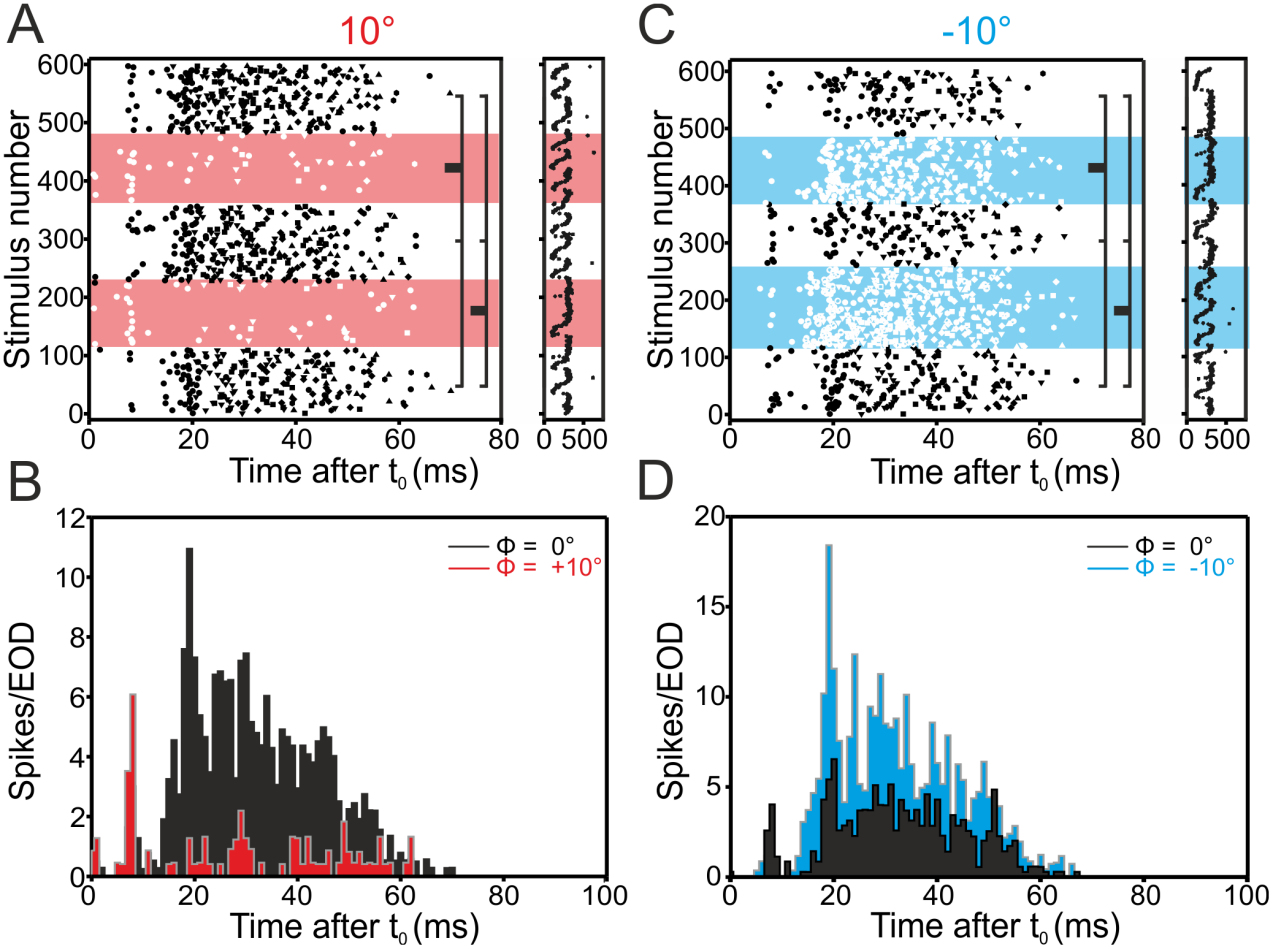
Example for an E-cell’s response in the dorsolateral zone to +10° (A, B) and -10° (C, D) phase-shifts. This cell responded with a reproducible de- (+10°) and increase (-10°) of its rate to the phase shifts, whereas first-spike latency was not systematically altered. Here and in the following figures four panels (A-D) are shown. **A, C**. Raster plots showing the change in spiking when switching from the undistorted (0°) to a phase-shifted (+ or -10°) EOD. Responses to phase shifted EODs are visualized by the coloured background. Significant differences between undistorted and phase-shifted conditions are indicated by the lines to the right (Kruskal-Wallis test with Dunn’s post-hoc analysis, *alpha* = 0.05). The raster plots on the right side of panels A and C depict the duration of the EODC for the corresponding raster plots. Note that EODC intervals were irregular and longer than the time at which spikes occurred. For better visualisation the raster plots are thus shown to match the longest interval after time zero at which spikes occurred in a given cell. **B, D**. Peri-stimulus time-histograms (PSTH) summarizing the data shown in A and C, phase shifts are plotted in colour, undistorted EOD-data in black. See S1 File for data.

We first report results obtained in the DLZ, where neurones are known to be responsive to phase-shifted stimuli. As expected, E-cells of the DLZ decreased the firing rate for positive phase shifts (N = 6 / 9) and increased their firing rate in response to negative phase shifts (N = 7 / 7, Table 1). This is shown for an exemplary cell in Fig 2, while the population data is shown in Fig 6B.

**Fig 3:**
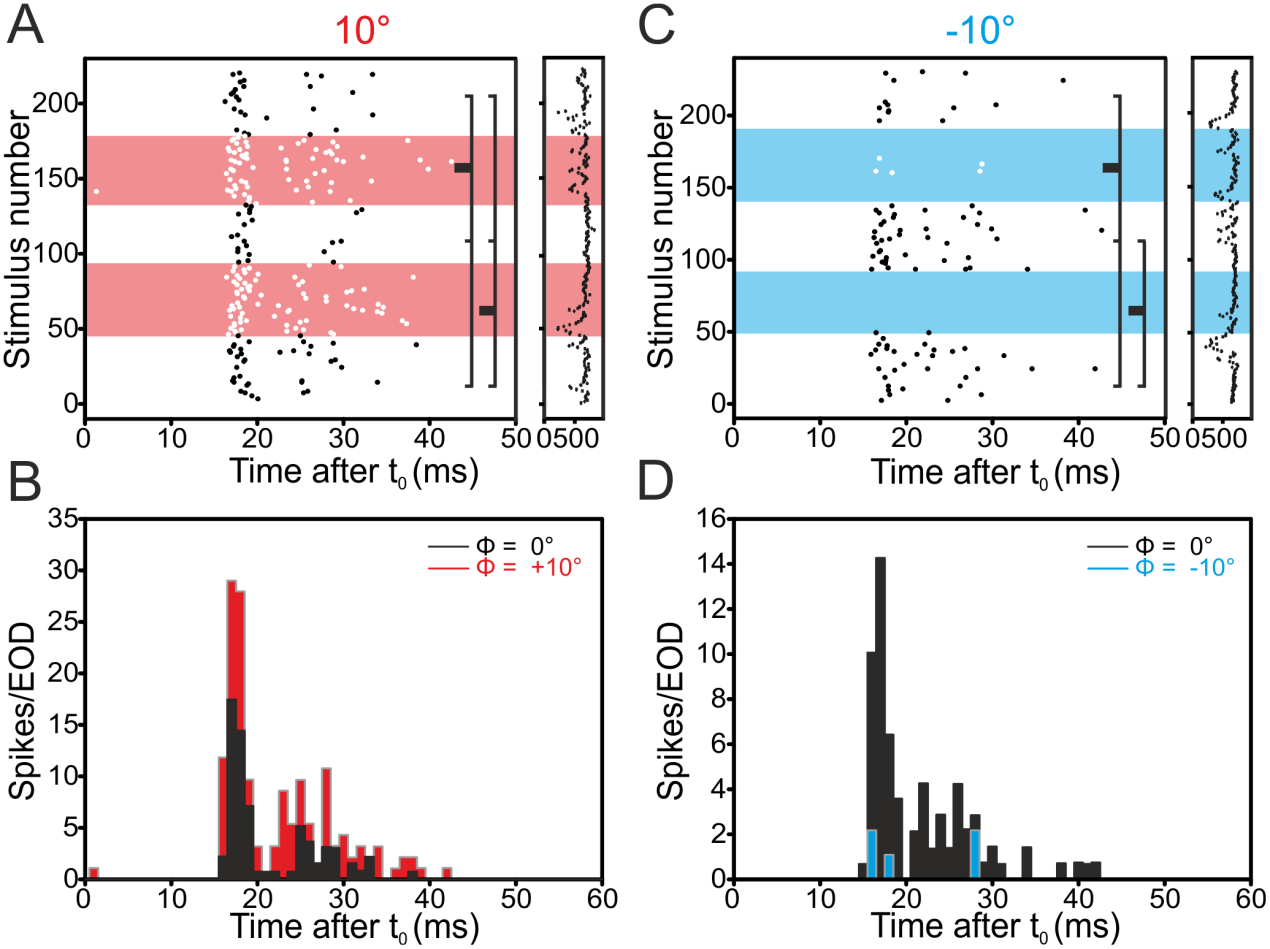
Example for an I-cell’s response in the dorsolateral zone to +10° (left, A-B) and -10° (right, C-D) phase-shifts. This cell responded reproducibly with an in- (+10°) or decreased (-10°) rate to the phase shifts, whereas first-spike latency was not altered systematically. For the full legend to the panels, refer to Fig 2.

As expected, I-cells in the DLZ increased the firing rate in response to positive shifts (N = 3 / 5) and decreased their rate in response to negative shifts (N = 3 / 5, see Figs 3 and 6 and Table 1).

In summary, our data on DLZ neurons corroborate published studies [20,31], which further indicates that our stimulation conditions were comparable to those used in these studies.

### Responses to phase-shifts in the MZ

**Fig 4:**
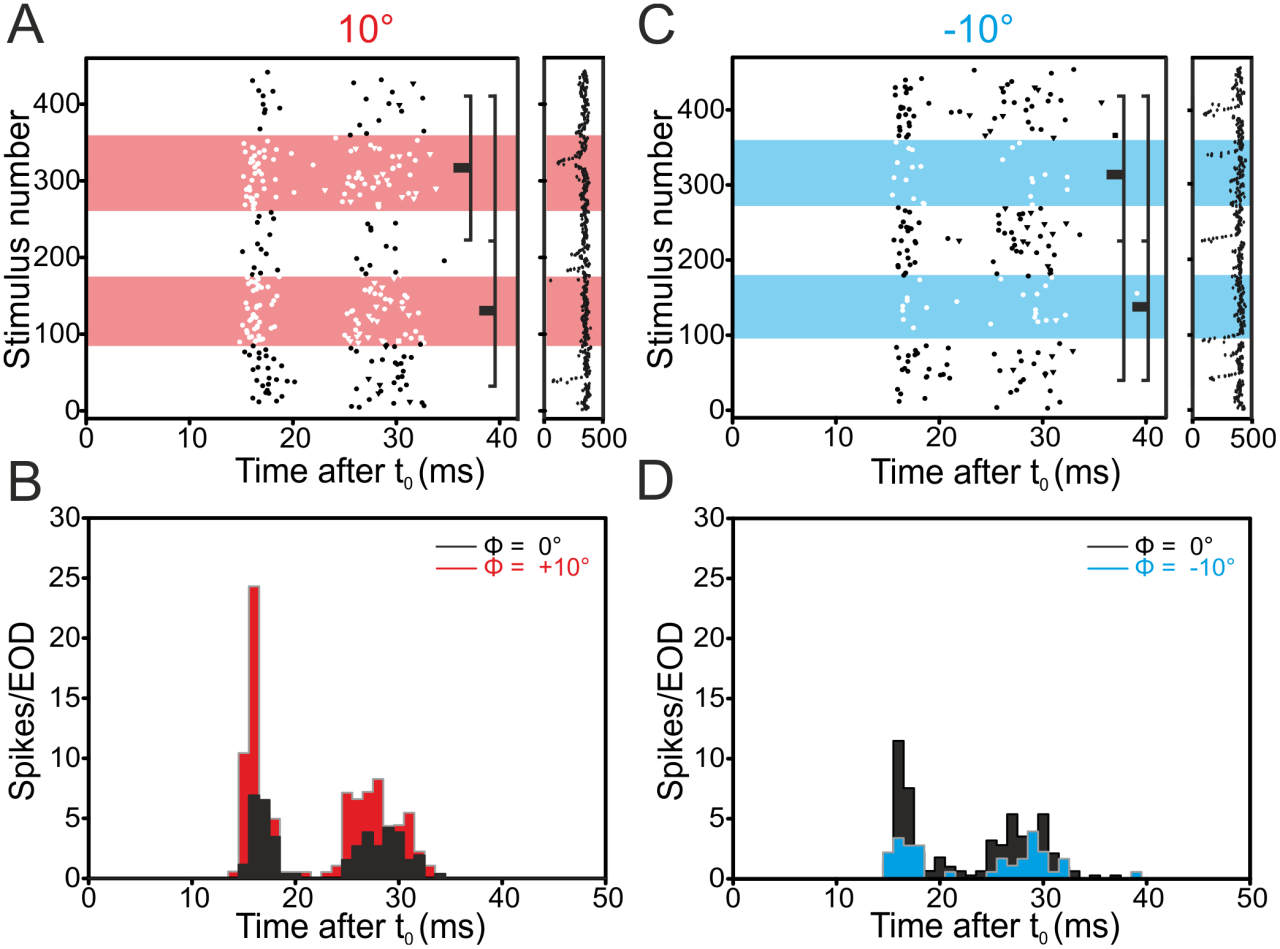
Example for an E-cell’s response in the medial zone to +10° (A, B) and -10° (C, D) phase-shifts. This cell responded reproducibly with an in- (+10°) or decreased (-10°) rate to the phase shifts, whereas first-spike latency was not altered significantly. For the full legend to the panels, refer to Fig 2. See S1 File for data.

In the following we report results on phase-shifted stimuli in the medial zone, where previous studies had not found evidence of phase-sensitivity. Three out of nine E-cells recorded in the MZ increased their firing rate in response to positive phase shifts in a reproducible manner (see Figs 4 and 6 and Table 1).

**Fig 5:**
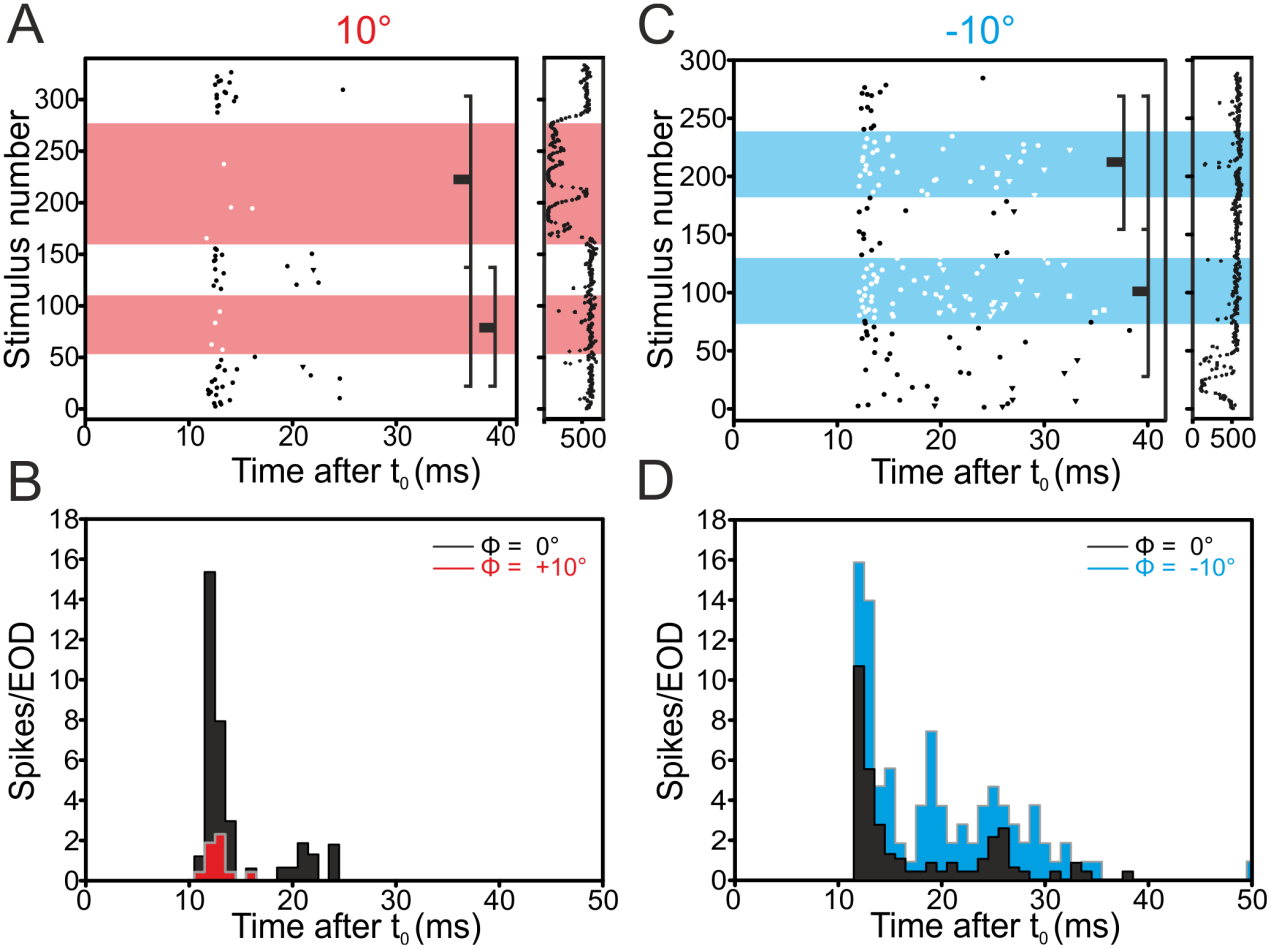
Example for an I-cell’s response in the medial zone to +10° (left, A-B) and -10° (right, C-D) phase-shifts. This cell responses reproducibly with a de- (10°) or an increased (-10°) rate to the phase shifts, whereas first-spike latency was not altered (not shown). For the full legend to the panels, refer to Fig 2. See S1 File for data.

**Fig 6:**
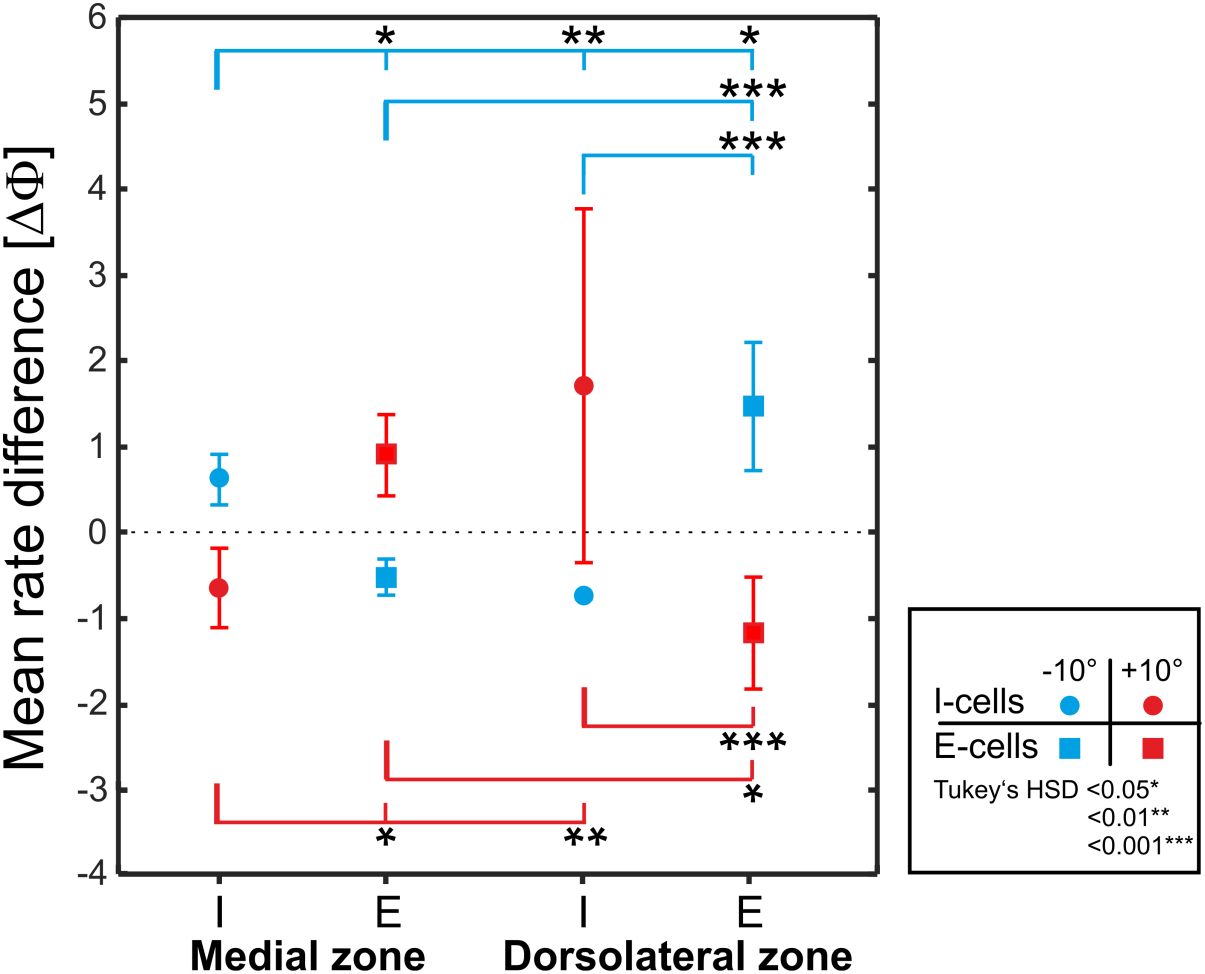
Summary graphs comparing the mean differences of I- and E-cells to phase-shifts in the medial and dorsolateral zone of the ELL. Solid symbols show data from cells the DLZ and open symbols show data from cells in the MZ with circles indicating data from I-cells and squares indicating data from E-cells. Responses to positive phase shifts are marked in red, while responses to negative shifts are marked in blue. Error bars represent standard deviations. Note that similar cell types respond oppositely in both zones. See S1 File for data.

Negative phase shifts led to a reproducible reduction of the firing rate in three out of nine E-cells tested (Figs 5 and 6). In I-cells, we found that eleven out of 20 cells decreased the firing rate reproducibly when subjected to positive phase-shifts (Figs 5 and 6 and Table 1). When stimulated with negative phase shifts, six out of these 20 cells increased their firing rates reproducibly.

### Comparison between zones

A 2-way analysis of variance with the main effects of zone (MZ and DLZ) and cell-type (E- and I-cells) was performed to compare the responses to phase shifts between zones and cell types. This revealed the presence of disordinal main effects (F-ratios for +10° shifts: Zone: F(1,19) = 0.12, p = 0.73; cell-type: F(1,19) = 2.75, p = 0.11; F-ratios for -10° shifts: Zone: F(1,15) = 1.57, p = .22; cell-type: F(1,15) = 4.59, p = 0.048) with significant interaction (F(1,19) = 30.68, p < 0.001) for +10° phase shifts and F(1,15) = 45.38, p < 0.001 for -10° phase shifts). Hence we focussed our analysis on the interaction effects, which were analysed using Tukey’s HSD post hoc test (Fig 6). As expected, E- and I-cells of the DLZ differed in their responses to phase shifted EODs both for negative and positive phase-shifts with E-cells responding to negative phase-shifts with an increase and I-cells with an decrease and vice versa for positive phase shifts (Tukey’s HSD, p < 0.001, Fig 6). The opposite effect was found for E- and I-cells in the medial zone (Tukey’s HSD, p < 0.05, Fig 6). A comparison between the zones confirmed that a given cell-type of the MZ will respond in a manner opposite to the same cell-type’s response in the DLZ (see Fig 6). This indicates that the phase sensitivity of neurones in the medial zone is not due to a direct (peripheral) input from the B-cells to this zone.

### Anatomy

In order to investigate how phase-sensitivity is conveyed from the DLZ to the MZ, we traced the connectivity between zones following tracer injections after either an electrophysiological experiment (N = 9) or, in four cases, in fish specifically injected for this purpose. In all cases injection sites were found in the zones targeted during the experiment, i.e., discrimination between zones based on our macroscopic and physiological parameters was correct.

**Fig 7:**
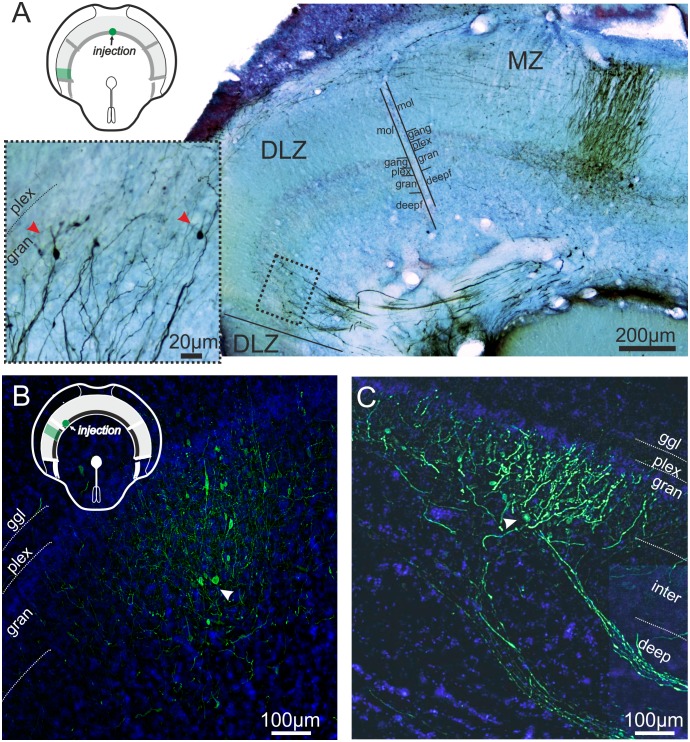
Anatomical data on interzonal connectivity. **A**. Low-magnification photomicrograph showing an ELL cross-section following a biocytin injection in the medial part of the medial zone. The section has been processed following the DAB procedure and counter stained with cresyl violet. The three zones are indicated on the slide and the different layers of the zones are indicated by the black lines at the borders between the MZ and DLZ. Note that the commissural projection connects the medial and dorsolateral zone such that the medial parts of the MZ connect with the lateral part of the DLZ. Likewise the lateral MZ is connected with the medial DLZ (see B, C). As the dorso-ventral topography of the sensory surface is represented along the medio-lateral axis in the MZ and the lateral to medial axis in the DLZ, this shows that somatotopically corresponding zones of the maps are connected. The higher magnification inset on the left shows the area surrounded by the stippled line in the DLZ. The red arrows point towards two retrogradely labelled somata in the granular layer. Abbreviations: deepf, deep fibre layer; gang, ganglionic layer; gran, granular layer; mol, molecular layer; plex, plexiform layer. **B**. Photomicrograph showing an injection site in the lateral part of the MZ using neurobiotin 488 (shown in green) and a fluorescent Nissl counterstain (blue). **C**. Interzonal projection to the DLZ originating from the injection shown in B. Note that in addition to the strong labelling of fibres some weakly stained large somata are present in the interzonal layer. The schematic inset in A and B shows a cross-section of the medulla with the injection site and the region where the close-up were taken indicated in green. deep, deep fibre layer; ggl, ganglionic layer, gran, granular layer, inter, intermediate layer; plex, plexiform layer.

As expected [32,40], injections in either the DLZ or MZ labelled homotopical projections to the other zone, i.e., the zones are interconnected and these connections maintain the topography between the maps (Fig 7), as originally shown by Bell and colleagues [41,42]. At present two cell types have been found to form interzonal connections, the Large multipolar intermediate layer cells (LMI) projections [43] and the interzonal cell [44]. Both cell types conserve topographic connections between zones where the interzonal cells terminate in the superficial and deep granular layers, while the axons of the GABAergic LMI cells terminate in the superficial granular layer. While we observed retrogradely labelled somata in the granular layer (Fig 7A) as well as larger somata in the intermediate layers (arrowhead Fig 7C), stainings were either too incomplete or dense to identify cell types. However, our data confirms the presence of a substantial homotopic interzonal connection, which we propose is the most parsimonious source of the phase-sensitivity described here for the cells of the MZ zone. Future studies are required to determine the detailed source as well as the connectivity between the zones to establish how the differential responses of E- and I-cells of the MZ and DLZ arise.

## Discussion

The independently evolved mormyroid and gymnotiforme weakly electric fish share a topographically organized representation of different electrosensory features in the hindbrain. In Gymnotiform fishes this parallel processing is achieved through differences in the spatiotemporal tuning properties of secondary neurones, whereas in Mormyrids this parallel processing already begins with two non-convergent sensory inputs from differently tuned electroreceptors. The observation that Mormyrids can distinguish complex impedances has led to the hypothesis that this may be based on a subtractive comparison of the phase and amplitude pathway input. A pioneering study found no evidence for the required convergence of the two sensory streams at the level of the ELL [31]. This is surprising given the significant interzonal connections between the two maps in the ELL [39,45,46]. Processing of capacitive information is particularly important for sensory input originating from the foveal chin appendix [32]. We here investigated to which degree the parallel sensory streams are kept separate downstream from the electroreceptors by specifically investigating the chin appendix region of the ELL.

Our results show that neurons from both the MZ and DLZ were affected by artificially generated phase-shifted stimuli. Importantly, phase-shifted EODs had the same peak-to-peak amplitude and power-spectral density as the non-shifted EODs (Fig 1) and only differed in the P/N-ratios. While this would not occur under natural conditions, it enabled us to selectively alter the response of B-cells. Any difference in neuronal response to shifted and non-shifted EODs are hence attributable to a waveform-sensitivity of the recorded neuron.

Our results for neurons in the DLZ corroborate published data [31], showing the expected decrease of the firing rate in I-cells and an increase in E-cells in response to negative phase shifts. Our study unveiled a fundamental difference to prior studies with respect to the medial zone. This zone receives only A-primary afferent input, and hence should not be responsive to phase shifts below 30° [25]. While prior studies did not find an effect of phase-shifted stimuli for neurons of the MZ that process sensory input form the trunk [24,31], our recordings of MZ-neurons that receive sensory input from the chin appendix showed that about half of all cells responded to phase-shifted stimuli well below 30°. We specifically focused on the chin appendix because this appears to be a sensory fovea devoted to impedance analysis [32]. We therefore expected that evidence for interzonal processing should be obtained in this part of the ELL most clearly. A second modification with respect to the earlier studies concerns the timing of the stimuli. We matched the timing of the stimuli to match the EOD timing measured prior to each experiment. The delay between the motor-command signal and the EOD varied between 2.4 and 4.9 ms in different fish, whereas the previous studies used a fixed delay of 5 ms. As sensory processing in the ELL largely depends on the timing of the interaction of a centrally originating corollary input to the ELL with the sensory input [47], this discrepancy likely has a considerable influence on the observed responses. It remains to be tested whether similar results might be obtained for neurones of the MZ that process sensory input from the trunk.

We suggest that the phase-sensitivity of the MZ is based on interzonal input. The two known cell types known to make interzonal connections both will be sensitive to a mismatched timing of corollary and sensory input [44]. Thus, it remains to be shown if the previous lack of evidence for phase-sensitivity is specific to the trunk regions of ELL, or rather related to the timing mismatch in earlier studies. For the responses of the DLZ the cause of the differential responses of E- and I-cells is the connectivity within this zone: negative phase shifts lead to an increased activity at reduced latency in the mormyromast B-cell primary afferents. These terminate on granular cells that make inhibitory synapses with I-cells (e.g. LG and MG1 cells) and are assumed to make excitatory synapses with E-cells (e.g. LF- and MG2 cells) [48]. Phase-shifts thus increase the inhibition of I-cells and increase the excitation of E-cells. This is in agreement with both our and the aforementioned studies. Future studies are required to address the cells and connectivity between the two zones. Our labelling confirmed the presence of reciprocal interzonal connections. Hence it remains to be investigated if and how the medial zone influences responses of the dorsolateral zone.

We here have reported results using artificial stimuli that were designed to deliver very local sensory activation at the periphery. The efferent neurones of the ELL are known to have centre-surround organisation [49,50], and it remains to be shown if centre and surround contribute differently to the phase sensitivity of neurones in the medial zone. Furthermore, under natural conditions, the sensory input consists of a global stimulation of the full array of electroreceptors. In a subset of cells we also tested the effect of global stimuli. However, as we only were able to do so in a total of eleven cells, of which 3 responded reproducibly to local shifts of the stimulus phase, we could not systematically analyse this data here. It should be noted however, that two neurones of the MZ that were classified as phase-sensitive also responded to globally shifted stimuli. His may suggest that the mediated phase-sensitivity is dominated by a cells centre, assuming that our local stimuli did selectively stimulate the centre. It has been shown that responses to global stimulation can differ considerably from responses to local stimuli such as the ones used in our present study [51]. If future studies confirm that phase sensitivity in the medial zone does not differ between local and global stimulation, this may further suggest that feedback from higher order electrosensory centres like the preeminential nucleus [52], are not involved in mediating this phase sensitivity.

In summary, our data are a first step to understand how weakly electric Mormyrid fish analyse their comparatively simple sensory environment through parallel processing. While we did not find “capacitance” encoding units in the ELL, we have shown that the parallel information is used to enhance the contrast between the two sensory streams at higher stages. Merging of these optimized ELL outputs most likely takes place at the torus semicircularis. To better understand the function of the two maps, future studies should aim to reveal the spatiotemporal properties of the dorsolateral zone to investigate if feature-optimized encoding schemes as reported for the three maps in Gymnotiform weakly electric fish (e.g., 50–52) exist in Mormyrid parallel maps as well.

## Supporting information

S1 File Data tables to reproduce Figs 2–6. The supporting information is provided in form of nine separate files compressed in a single RAR-archive. Files named Fig2a.txt to Fig5b.txt contain the data to reproduce figures 2–5, while the file Fig6.txt contains the data to reproduce the data shown in figure six for all cells as well as the cells that responded in a reproducible manner to phase shifts. In all files the first row indicates what kind of data is being shown in the columns. Data in columns named AP1 to AP14 (Fig2a.txt to Fig5b.txt) show the latency of spikes in response to the EOD mimic in seconds.
(RAR)
